# Gas Plasma Combination Therapies—Promises from Preclinical Oncology Research

**DOI:** 10.3390/antiox14091055

**Published:** 2025-08-27

**Authors:** Lingyun Yu, Julia Berner, Alice Martinet, Eric Freund, Debora Singer, Thomas von Woedtke, Klaus-Dieter Weltmann, Steffen Emmert, Ramona Clemen, Sander Bekeschus

**Affiliations:** 1 Department of Dermatology, Venerology, and Allergology, Rostock University Medical Center, Strempelstr. 13, 18057 Rostock, Germany; 2 ZIK *plasmatis*, Leibniz Institute for Plasma Science and Technology (INP), Felix-Hausdorff-Str. 2, 17489 Greifswald, Germany; 3 Department of Neurosurgery, Medical University of Vienna, Waehringer Guertel 20, 1070 Vienna, Austria; 4 Institute for Hygiene and Environmental Medicine, Greifswald University Medical Center, Sauerbruchstr., 17475 Greifswald, Germany

**Keywords:** CAP, chemotherapy, drugs, electrochemotherapy, immunotherapy, ionizing radiation, LTP, NTP, plasma medicine, photodynamic therapy, radiotherapy

## Abstract

The absent decline in cancer mortality rates is primarily due to moderate therapeutic efficacy and intrinsic or acquired tumor cell resistance toward treatments. Combining different oncology treatments increases therapy success and decreases the chance of refractory tumor cells. Therefore, combination cancer treatments are the principal paradigm of 21st-century oncology. Physical modalities such as radiotherapy have a long-standing tradition in such combination treatments. In the last decade, another physical principle emerged as a promising anticancer agent: cold gas plasma. This partially ionized gas, operated at about body temperature, emits multiple bioactive components, including reactive oxygen and nitrogen species (ROS/RNS). This technology’s multi-ROS/RNS nature cannot be phenocopied by other means, and it capitalizes on the vulnerability of tumor cells within metabolic and redox signaling pathways. Many cancer models exposed to mono or combination gas plasma treatments have shown favorable results, and first cancer patients have benefited from cold gas plasma therapy. The main findings and proposed mechanisms of action are summarized. Considering the specific application modes, this review identifies promising gas plasma combination therapies within guideline-directed treatment schemes for several tumor entities. In conclusion, gas plasmas may become a potential (neo)adjuvant therapy to existing treatment modalities to help improve the efficacy of oncological treatments.

## 1. Intoduction

Despite progress in diagnostic techniques and therapeutic approaches in oncology, the global cancer burden is still increasing and constitutes the leading cause of death worldwide [1]. Although cancer mortality rates have gradually improved among Western countries, cancer incidences are rising for the majority of malignancies. Moreover, the efficacy of many oncological treatments is insufficient and steadily results in therapy failures. Intrinsic or acquired resistances further lead to tumor relapses that cause poor patient prognosis. Multimodal combination approaches are proposed as the most promising innovation in the fight against cancer since they comprise the simultaneous or sequential administration of multiple therapy options, exploiting different mechanisms of action to achieve an augmented efficacy. Additionally, the risk of tumor resistance is reduced, and the severity of toxic side effects can be alleviated since a combination of complementary treatment techniques can sensitize tumor cells, lowering the required therapeutic dosage of the second treatment to obtain the same or stronger antitumor effect [2,3]. For example, a phase II clinical trial investigating a combinatory approach of curcuminoids with concurrent chemotherapy demonstrated improved efficacy paralleled by reduced adverse events and increased quality of life in patients compared to monotherapy [4].

Cold physical plasma, an oxidative stress-inducing multicomponent system, has been suggested as a novel adjuvant therapy tool as it has shown promising potential for anticancer treatment [5] and even more powerful tumor toxicity in combination with other therapeutic options like radiotherapy, chemotherapy, or nanoparticles [6,7,8]. Due to its highly reactive nature, gas plasma can prime tumor cells for additional treatments and elicits cell physiological changes like the upregulation of drug transporters or immunogenic cell death markers, which can lead to enhanced toxicity of co-therapies by elevated intracellular accumulation of chemotherapeutic agents (e.g., doxorubicin) or efficacy of immunotherapies, respectively [8,9]. Simultaneously, it shows prominent tumor selectivity that enables evidently good tissue tolerability and low side effects, rendering gas plasma a promising technology to sensitize cancer cells in the context of combination therapies and thus revolutionizing multimodal anticancer concepts.

This review summarizes the main findings of combining cold physical plasma with other oncological approaches and current preclinical progress across several cancer types. Furthermore, the translation of such combination strategies in prospective medical applications, potential future objectives, and limitations that need to be addressed for successful clinical implementation are discussed.

## 2. Gas Plasma Technology

**Figure 1:**
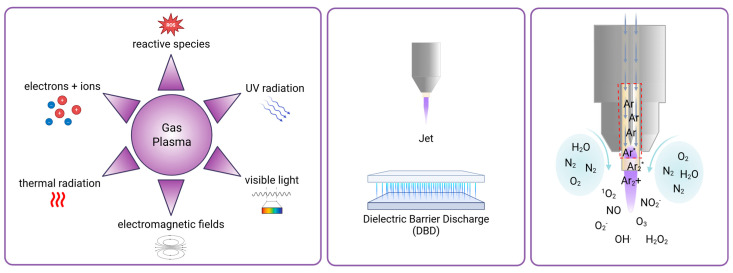
Gas plasmas’ main biologically active components, device types, and exemplary principle of ROS production. Gas plasma is composed of various physical and chemical gas plasma components with biological activity (**left**). Gas plasma for medical applications is commonly generated with either atmospheric pressure plasma jets, in which a feed gas (e.g., helium or argon) flowing through a nozzle is excited by applying high voltage, or with dielectric barrier discharge (DBD) devices, where ambient air is excited between two electrodes. Representative drawings of the two device types are shown here (**middle**). Reactive species causing biological effects like singlet oxygen, superoxide, ozone, nitric oxide, nitrate, hydroxyl radical, and hydrogen peroxide are generated by the reaction of excited gas molecules (e.g., argon gas in a jet device, red square) with oxygen, nitrogen, and water molecules from ambient air (**right**).

Gas plasma, also known in physics as physical plasma, is a partially ionized gas composed of various bioactive components like reactive species, light (VIS, UV), thermal radiation, and electromagnetic fields (Figure 1). It is generated by applying external energy, usually in the form of an electric field, to excite gas molecules [10]. The applied electric field energizes electrons that can collide with gas molecules of the ambient air, creating new charged particles. Leap innovations, a few decades ago, facilitated the generation of plasmas at atmospheric pressure into the ambient air while remaining at about body temperature. This enabled the use of cold plasma for biomedical and clinical applications [11]. Due to the potent antimicrobial activity of gas plasma exposure [12,13], the technology was soon tested for the treatment of chronic, non-healing wounds and ulcers, as these are often heavily infected with microorganisms of different kinds [14,15]. Intriguingly, early clinical investigations were successful in reducing microbial burden in wounds and facilitated their healing [16,17], leading to the approval of several medical gas plasma devices in Europe for dermatology applications [18]. Thus, multiple risk assessment studies have demonstrated and validated the safety and good tissue tolerability of this therapeutic tool. Due to its high tumor selectivity [19,20,21], the plasma application causes merely low side effects, being primarily restricted to the local treatment area. No mutagenic, genotoxic [22], or long-term side effects were observed in animal experiments [23], and irritation assays using the in ovo HET-CAM egg model revealed no significantly higher irritative potential compared to conventional therapeutic agents, e.g., cisplatin, but instead a notably better reversibility of induced irritative pattern [24].

Standard methods to generate such gas plasmas for clinical use include dielectric barrier discharge (DBD) [25], where electrical discharge occurs in ambient air between two electrodes separated by an insulating dielectric barrier, and jet discharge, where a working gas (such as helium or argon) flows through a nozzle or capillary and generates a plasma plume by applying high voltage [26] (Figure 1). Reactive oxygen and nitrogen species (ROS/RNS) of various types produced by gas plasmas were found to be significant components mediating biological effects in gas plasma, independent of the gas plasma device geometry being jet-like or DBDs [27]. Major ROS are, for instance, superoxide (O_2_^−^), singlet oxygen (^1^O_2_), ozone (O_3_), hydroxyl radical (•OH), and hydrogen peroxide (H_2_O_2_). Major RNS are, for example, nitric oxide (NO) and nitrite (NO_2_^−^) [28] (Figure 1). As most RNS contain reactive oxygen, ROS/RNS will be abbreviated as ROS from hereon. Next to its supportive properties for wound healing, it was shown that gas plasma is also able to confer cytotoxicity based on dose-dependent opposing effects, which are explained by the concept of hormesis [29]. In cells and tissues, it is known that ROS can lead to oxidative eustress and distress [30]. Oxidative eustress is essential to cell viability, differentiation, and proliferation [31]. On the contrary, excess ROS apparent in oxidative distress causes cell damage or even death by triggering downstream effects, including DNA strand breaks, oxidation of proteins and lipids, resulting in activation of apoptotic pathways [32]. The potential of gas plasma to treat cancer was shown in recent years for a number of different tumor entities, including breast cancer, colorectal cancer, glioblastoma, leukemia, lung cancer, melanoma, and squamous cell carcinoma (SCC) in vitro and in vivo, as reviewed before [33].

The induced cell death modality of apoptosis is mainly reported to be the cause of gas plasma-mediated anticancer effects, visible in, e.g., caspase 3 and 7 activation [34], mitochondrial membrane potential loss [35], or histone 2AX phosphorylation (yH2AX) [36,37]. Nevertheless, further types of cell death were found to be induced, such as ferroptosis [38,39], pyroptosis [40], and autophagy [41]. Another mode of action observed in gas plasma-mediated cancer cell death is the modulation of immunogenicity. Hallmarks of immunogenic cell death (ICD), like ATP secretion [42] or HMGB1 externalization [43,44], have been shown to be induced upon plasma treatment in vitro and in vivo. Despite broad preclinical evidence, plasma cancer treatment is still lacking clinical studies. So far, only five clinical trials investigating gas plasma for the treatment of cancer or precancerous lesions registered on ClinicalTrials.gov have been completed (Table 1). The completed trials on plasma treatment of cervical intraepithelial neoplasias (CIN) [45], anal intraepithelial neoplasias (AIN) [46], actinic keratosis lesions [47], and combination with tumor resection [48] could already show promising anticancer actions of gas plasma while being safe without serious adverse events.

## 3. Combination Treatment with Gas Plasmas in Experimental Models

Gas plasma emerged as a cancer treatment and has been proven effective. It has broadened its application via various therapies in oncology. Furthermore, cold gas plasma exposure can display local and/or systemic properties. The former indicates that gas plasma could be directly used locally on tumor entities like melanoma, while the latter points to gas plasma-driven immune-related mechanisms [5] and/or gas plasma-treated liquid (PTL) used to flush larger body cavities such as the peritoneum [49]. Several in vitro and in vivo studies (Table 2) combining gas plasma technology with other systemic or local anticancer approaches are available, and significant findings are summarized in the following sections.

Although surgery serves as the preferred first-line therapy, particularly for early or mid-stage tumors, advanced disease stages require multimodal therapies to eradicate local malignancies and metastatic lesions [50,51]. Systemic therapies have the benefit of reaching primary tumors and metastases at almost all body sites through the blood circulation. Still, they are often associated with severe side effects, such as nausea, vomiting, and impaired immunity [52]. In contrast, local therapies directly impose their action in an often spatiotemporally controlled manner onto the treatment sites. Even if the clinical discipline of oncology has evolved multidisciplinary and partially highly successful treatment regimes, existing or new challenges arise with those, such as drug resistance triggering therapy failures and long-term treatment duration, as well as adverse events reducing a patient’s quality of life [53], continuously motivating the search for innovative anticancer treatments. Implementation of cold physical plasma as a medical adjuvant could bridge known shortcomings of insufficient treatment options in a synergistic or additive manner. Gas plasma-induced oxidation of cell membranes could increase the membrane permeability, promoting uptake of applied therapeutic agents [54,55] and sensitization of tumor cells for the following treatments [56]. Furthermore, a plethora of redox-regulated signaling pathways are initiated that can lead to augmented expression of drug-relevant transporters, which in turn facilitate the enhanced intracellular accumulation of chemotherapeutics. By stimulating immunogenic cancer cell death, plasma additionally mounts the antitumor immunity, not only tackling tumor metastasis but also recurrences. Patients could tremendously benefit from the integration of gas plasma in multimodal concepts, as it has the potential to optimize therapeutic efficacy, improve therapy outcomes, and decrease the required dose of toxic treatment approaches, e.g., chemotherapy, mitigating undesired side effects.

**Table 2:** Studies on combining gas plasmas with anticancer treatment modalities. Anticancer treatments combined with gas plasma treatment are sorted alphabetically. BT = biologicals (e.g., virotherapy, hormone therapy, and non-checkpoint antibody therapy); CT = chemotherapy; EA = experimental agents; ECT = electrochemotherapy; HT = hyperthermia; IT = immunotherapy (including checkpoint antibody therapy); NP = nanoparticle; PDT = photodynamic therapy; PEF = pulsed electric fields; RT = radiotherapy; and TT = targeted therapy.

Combined Anticancer Treatment	Type of Cancer	Agent/Clinically Relevant	Gas Plasma Source	Study Model	Additive/Synergistic Effect	Ref
BT	Melanoma	Cell starvation/yes	kINPen (Ar)	In vivo: Syngeneic	Yes	[57]
BT	Breast	Melittin/yes	kINPen (Ar)	In ovo: TUM-CAM	Yes	[58]
CT	Melanoma	Cyclophosphamide/no	Jet (He)	In vivo: Syngeneic	Yes	[59]
CT	Melanoma	Dacarbazine/yes	Jet (Ar)	In vivo: Syngeneic	Yes	[60]
CT	Melanoma	Bleomycin/yes Dacarbazine/yes Paclitaxel/yes	Jet (Ar)	In vitro	Yes	[61]
CT	Melanoma	Doxorubicin/yes Epirubicin/yes Oxaliplatin/yes	kINPen (Ar)	In vitro	Yes	[8]
CT	Melanoma	Doxorubicin/yes	DBD	In vitro	Yes	[62]
CT	OSCC	Cisplatin/yes	P500-SM (Ar)	In vitro	Yes	[63]
CT	HNSCC	Cisplatin/yes	SMD	In vitro	Yes	[64]
CT	Breast	Doxorubicin/yes	Jet (He)	In vitro	Yes	[65]
CT	Breast	Doxorubicin	Jet (Ar)	In vitro	Yes	[66]
CT	Breast	Paclitaxel/yes	DBD	In vitro	Yes	[67]
CT	Glioma	Temozolomide/yes	Jet (He)	In vitro	Yes	[68]
CT	Glioma	Temozolomide/yes	kINPen (Ar)	In vitro	Yes	[69]
CT	Glioma	Temozolomide/yes	DBD	In vitro	Yes	[70]
CT	Glioma	Temozolomide/yes	SMD	In vitro	Yes	[71]
CT	Glioma	Topotecan/no	Glow charge/spark discharge (Air)	In vitro	Yes	[72]
CT	Endometrial, Gastric	Cisplatin/yes	Unknown (Ar)	In vivo: Xenograft	Yes Yes	[73]
CT	Liver	Cisplatin/yes	DBD	In vitro	Yes	[74]
CT	Pancreas	Gemcitabine/yes Cisplatin/yes	kINPen (Ar)	In ovo: TUM-CAM	Yes Yes	[75]
CT	Pancreas	Gemcitabine/yes	Plasma gun (He)	In vitro	Yes	[76]
CT	Bladder	Cisplatin/yes Methotrexate/yes Adriamycin/yes paclitaxel/yes	Jet (He)	In vitro	Yes	[77]
CT	Ovary	Cisplatin/yes	Jet (Ar)	In vitro	Yes	[78]
CT	Prostate (Bone Metastasis)	Doxorubicin/yes	kINPen (Ar)	In vitro	Yes	[79]
CT	Ewing Sarcoma	Doxorubicin/yes Vincristine/yes	kINPen (Ar)	In vitro	Yes Yes	[80]
CT	Ewing Sarcoma	Methotrexate/yes Cisplatin/yes	kINPen (Ar)	In vitro	Yes	[37]
CT	Osteosarcoma	Salinomycin/no	Jet (He)	In vivo: Syngeneic	Yes	[81]
EA	Melanoma, SCC	Sm837/no, IS112/no	kINPen (Ar)	In vivo: Xenograft	Yes	[82]
EA	Melanoma	ADDA 5/no	kINPen (Ar)	In vitro	Yes	[83]
EA	Melanoma	Curcumin/no	Jet (Ar)	In vitro	No	[84]
EA	Melanoma	Salinomycin/no	Jet (Ar)	In vitro	Yes	[81]
EA	Breast	Pluronic F127/no	Jet (He)	In vivo: Syngeneic	Yes	[85]
EA	Non-Small Cell Lung Cancer	Chloroquine/no	Jet (He)	In vitro	Yes	[86]
EA	Glioma	Auranofin/no	kINPen (Ar)	In vivo: Syngeneic	Yes	[87]
EA	Glioma	Vitamin C/no	kINPen (Ar)	In vivo: Xenograft	Yes	[88]
EA	Glioma	Pyrazolopyrimidinone/no	DBD	In vitro	Yes	[89]
EA	Acute Lymphoid Leukemia	Sulfasalazine/no	PN-120TPG (Ar)	In vitro	Yes	[90]
ECT	Melanoma	ECT bleomycin/yes	Jet (Ar), DBD	In vivo: Syngeneic	Yes	[91]
ECT	Fibrosarcoma	ECT bleomycin/yes	PMJ (He)	In vivo: Syngeneic	Yes	[92]
HT	Non-Small Cell Lung Cancer	Hyperthermia/yes	Unknown (Ar)	In vitro	Yes	[93]
IT	Melanoma	Anti-PD-L1/no	Jet (He)	In vivo: Syngeneic	Yes	[94]
IT and EA	Melanoma	Pembrolizumab/yes, A-1210477/no, Carvedilol/no, Cozymasei/no, SBI-0206965/no, Navitoclax/no	kINPen (Ar)	In vivo: Syngeneic	Yes	[95]
NP	Melanoma	Nanoparticle/no (Si, Ag, FeO, CeO_2_, TiO_2_, FeTiO_2_)	kINPen (Ar)	In vitro	Yes	[96]
NP	Melanoma	Anti-NEU gold nanoparticle/no	DBD	In vitro	Yes	[97]
NP	Melanoma	p-FAK gold nanoparticle/no	DBD	In vitro	Yes	[98]
NP	Melanoma	Anti-FAK gold nanoparticle/no	Unknown (Air)	In vitro	Yes	[99]
NP	Melanoma	Silver nanoparticle/no	pm-rf-APGD	In vitro	Yes	[100]
NP	Breast	Iron particle/no	Jet (He)	In vitro	Yes	[101]
NP	Breast	Nanoparticle/no	Unknown	In vitro	Yes	[102]
NP	Colon	Gold nanoparticle/no	Jet (He)	In vitro	Yes	[103]
NP	Glioma	Gold nanoparticle/no	DBD	In vitro	Yes	[104]
NP	Glioma	Silver nanoparticle/no	DBD	In vitro	Yes	[105]
NP	Glioma	Gold nanoparticle/no	Jet (He)	In vitro	Yes	[106]
NP	Glioma	Gold quantum dots/no	Jet (Air)	In vitro	Yes	[107]
NP	Non-Small Cell Lung Cancer	Iron oxide-based magnetic nanoparticle/no	Jet (He)	In vitro	Yes	[108]
NP and BT	Melanoma	Silymarin nanoemulsion/no	DBD	In vitro	Yes	[109]
NP and BT	Melanoma	Silymarin nanoemulsion/no	DBD	In vivo: Xenograft	Yes	[110]
NP and CT	Non-Small Cell Lung Cancer	Paclitaxel-loaded magnetic nanoparticles	Jet (He)	In vitro	Yes	[111]
NP and PDT	Melanoma	Photodynamic therapy (nanoparticle)/yes	Unknown	In vitro	Yes	[112]
PDT	Colon	Photodynamic therapy/yes	Jet (He)	In vitro	Yes	[113]
PDT	Glioma	Photodynamic therapy/yes	Jet (He)	In vitro	Yes	[113]
PDT	Non-Small Cell Lung Cancer	Photodynamic therapy/yes	Jet (He)	In vitro	Yes	[114]
PEF	Pancreas	Pulsed electric field/no	Jet (He)	In vitro	Yes	[115]
RT	Melanoma	Radiation/yes	kINPen (Ar)	In vitro	Yes	[116]
RT	Hepatoblastoma	Radiation/yes	Jet (Ar + O_2_)	In vivo: Xenograft	No	[6]
RT and TT	Breast	Radiation therapy/yes Olaparib/yes	Jet (He)	In vitro	Yes	[117]

### 3.1. Systemic Agents

#### 3.1.1. Chemotherapy

Chemotherapies are traditional anticancer treatments using synthetic or extracted elements, which will circulate through the body fluid by intravenous or oral medication and then enter cells to interfere with cellular metabolism. Chemotherapy is classified into alkylating agents, antimetabolites, hormones and antagonists, natural products, and miscellaneous [118], and it is known to mainly hamper DNA and RNA synthesis and disrupt mitosis [119]. Current gas plasma combinations with chemotherapy all show synergistic effects in at least one cell line. So far, clinically relevant chemotherapies investigated only involve three categories: alkylating agents, antimetabolites, and natural products.

In alkylating agents, cyclophosphamide combination points out that the synergistic effect is associated with activation of p53 and Bax/Bcl-2 markers by plasma via enhancing ROS levels [59]. Dacarbazine combination showed a synergistic effect in melanoma cells in vitro and in vivo [60] and an 18% increased anticancer effect [61] in glioblastoma multiforme (GBM) cell lines in vitro, but the regulation mechanisms were not unraveled yet. Oxaliplatin [8] does not display a pronounced synergistic effect in the melanoma cell line. Cisplatin combinations are widely investigated in seven studies (Table 2), showing combinatorial effects, but still focus on phenotypic effects such as cell death [63,64,75] or cell cycle arrest [75], lacking mechanistic investigations. For temozolomide (TMZ), gas plasma could improve GBM cell sensitivity towards TMZ chemotherapy in 06-methylguanine-DNA methyltransferase (MGMT)-positive TMZ-resistant cells [71]. Further, gas plasma augmented TMZ cytotoxicity by ROS-mediated DNA damage, visible in the induction of H2AX phosphorylation [68] and increased 8-hydroxy-2′-deoxyguanosine (8-OHdG) and 8-Oxo-2′-deoxyguanosine (8-oxodG) levels after inhibiting antioxidative glutathione (GSH)/glutathione peroxidase 4 (GPX4) signaling [69]. Regarding antimetabolites, methotrexate displays poor anticancer selectivity [77], and gemcitabine is initially being sought for combination with gas plasmas or plasma-treated liquids against pancreatic cancer [75,76], both of which have not yet revealed underlying mechanisms. Among natural products, salinomycin and plasma-treated infusion cooperatively promoted disruption of the mitochondrial network [81]. Further, a synergistic toxicity with doxorubicin was connected to improved drug uptake by *SLC22A16* upregulation [8] or decreased drug efflux via downregulation of drug-resistance-associated *ABCC1* (MRP1), *ABCB1* (MDR1), and *ABCG2* (BCRP1) upon plasma treatment [79]. Other natural products (bleomycin [61], paclitaxel [61,67,77], vincristine [80]) show a lack of mechanism exploration.

Based on the above studies, gas plasma could be an interesting tool for overcoming drug resistance, and, if applied locally to a tumor, it might decrease the dosage of chemotherapies. Notably, most of these studies focused on alkylating agents and natural products, and future research could also extend to other drugs. However, chemotherapy resistance and its side effects have emerged as prominent issues in patients, and it is necessary to pursue effective means to resolve them.

#### 3.1.2. Targeted Therapy

Targeted therapy primarily anchors at molecular loci via specific molecules binding, and is generally divided into two classes: small-molecule inhibitors and monoclonal antibodies (mAbs) [120]. Small molecules can be further categorized into tyrosine kinase inhibitors (TKIs), poly ADP ribose polymerase (PARP) inhibitors, proteasome inhibitors (PIs), and cyclin-dependent kinase (CDK) inhibitors [121]. Targeted therapy should be distinguished from immunotherapy, especially with regard to mAbs. According to their mechanism of action, mAbs can be broadly classified into two groups: those exerting antitumor effects by directly targeting tumor-related molecules (targeted therapy) and those modulating the immune system by checkpoint inhibition [122]. The latter will be discussed in the immunotherapy section.

In essence, commonly used targeted sites include HER (human epidermal growth factor receptor) 2, EGFR (epidermal growth factor receptor), and VEGFR (vascular-endothelial growth factor receptor) [123]. These sites can be targeted by mAbs, like trastuzumab [124] or cetuximab, or TKIs like sorafenib [125,126]. For instance, gas plasma-mediated toxicity has been shown in lung cancer cell growth through the VEGF/VEGFR2/RAS axis [127] and in hepatocellular carcinoma via enhanced autophagy and the EGFR/p-JUK/BIRC6/LC3B axis [128]. A combination of cetuximab and plasma was shown to inhibit the invasion/migration of oral squamous cell carcinoma (oSCC) cells through NF-kappaB signaling regulation [129]. Synergy between TKIs and oxidative stress was reported in a study using hydrogen peroxide in colorectal cancer [130], suggesting gas plasma to be an interesting approach to combine with targeted therapies. Interestingly, gas plasma-mediated ROS were also shown to impact the targeted tyrosine kinase proteins themself by introducing oxidative posttranslational modifications (oxPTMs), which might lead to altered structure and catalytic function [131]. If these modifications synergize with TKIs’ inhibitory effects or instead lower their effectiveness, it remains to be investigated. For CDK inhibitors, a combination of an indirubin derivative (KD87) with gas plasma-treated medium was shown to induce G2/M arrest and apoptosis via AhR pathway activation [132]. As a PARP inhibitor, olaparib shows a synergistic effect with plasma [117], but no further research was conducted to clarify the underlying pathway. Among PIs, plasma could increase the sensitivity of myeloma cells to bortezomib [133] to achieve an anticancer effect. Due to their overlap with the above-mentioned plasma combination approaches, TKIs and mAbs may serve as promising entry points for further investigations.

Next to synergizing mechanisms of action, drugs such as TKIs could be affected by gas plasma exposure, potentially altering their activity. This was seen in a study using 37 gas plasma-treated TKIs, showing mainly a reduction in TKI toxicity but also an increased toxicity for five compounds [134]. Such alterations can be induced by gas plasma-mediated oxidation of specific structures of the drug molecules, e.g., at the warhead groups necessary for the binding to the targeted kinase. Another interesting approach is the activation of prodrugs, which was, for example, shown for fenretinide via gas plasma-induced oxidation [135]. This way, gas plasma could be used as a tool to induce on-site activation of prodrugs, e.g., in superficial tumors, reducing off-target effects.

#### 3.1.3. Immunotherapy

Although gas plasma was first tested in cancer patients in the mid-2010s [136], clinical combination therapies of gas plasma and immunotherapy have not been reported (Table 2). In general, immunotherapies are classified as either passive or active [137]. Often, immunotherapies act via tumor-infiltrating lymphocytes (TILs), chimeric antigen receptor (CAR) T-cells, lymphokine-activated killer cells (LAK), cytokine-induced killer cells (CIK), and antibody-based immune checkpoint inhibition (ICI) like CTLA-4 and PD-1/PD-L1 [138]. It was previously proposed from preclinical models that gas plasma should not be used as monotherapy but instead combined with immunotherapy to improve anticancer effects [139,140]. A promising approach is to functionalize the tumor microenvironment (TME) by enhancing the local function of anticancer immunity, for instance, by inducing immunogenic cell death (ICD) and triggering the influx and activity of antitumor leukocytes [5]. For example, gas plasma-treated skin cancer cells showed a higher susceptibility to NK-cell-induced tumor cell lysis than untreated skin cancer cells [141]. Gas plasma treatment was further shown to enhance tumor infiltration with T-cells and dendritic cells (DCs) in treated tumors and in an abscopal way in vivo [9] and increase phagocytosis of cancer cells by DCs [142].

Two studies have demonstrated synergistic effects combining gas plasma treatment with ICI. A combination of gas plasma anti-PD-L1 in a syngeneic melanoma model in vivo resulted in improved tumor reduction and increased TILs [94]. This finding is supported by a second study combining pembrolizumab (anti-PD-1) for the first time with an improved atmospheric pressure argon plasma jet routinely applied in dermatology [95]. The underlying mechanism involved gas plasma-induced oxidative stress triggering ICD, characterized by the release of damage-associated molecular patterns (DAMPs), which consequently attracted antigen-presenting cells (APCs). No studies have been performed combining anti-CTLA-4 with gas plasma yet. Given the promising approaches stated above, more research is expected in combining immunotherapies with gas plasma technology in immuno-oncology.

#### 3.1.4. Nanoparticles

Nanoparticles have been considered promising since they were first proposed in 1959 [143]. Different from standard therapies, nanoparticles function as drug carriers and effective agents simultaneously, and their roles in cancer have been reviewed elsewhere before [144]. Their effectiveness depends on particle diameter, shape, types, elasticity, superficial structures, and chemical modifications [145,146]. Nanoparticles can be used in biomedicine for drug delivery and biological imaging due to their large surface-to-volume ratio, enabling the attachment of cargos like fluorophores, drugs, or antibodies, as well as a deep tissue penetration [147,148]. Generally, nanoparticles can enter cells through endocytosis and non-endocytosis-mediated passive penetration [149]. They can lower drug toxicity through enhanced permeability and retention (EPR) effects. In contrast, the safety of nanoparticles themselves remains controversial [150]. Encouragingly, reports suggest combined effects of gas plasma and nanoparticle exposure, including silymarin nanoemulsion and Pluronic F127 (Table 2). Interestingly, intracellular ROS generation was described for different types of nanoparticles, like metallic oxide NPs and silica particles [151], which could combine with gas plasma-generated extracellular ROS [28]. Combined toxicity was found in melanoma [96], while gas plasma exposure of the particles also affects the melanoma toxicity of the latter [100]. This was suggested due to a decreased nanoparticle size and modified surface structure in response to gas plasma exposure [105,152]. At the same time, elevated PLGA (Poly Lactic-co-Glycolic Acid) film hydrophilicity was indicated as an effector in improved combined toxicity [153], along with increased nanoparticle uptake via enhanced clathrin-dependent endocytosis [154]. On the cellular level, gas plasma may alter cell membrane permeability and nanoparticle accumulation [106,155]. Owing to multiple nanoparticle synthesis ways, nanoparticles can have cores and shells [102]. Thus, a drug–nanoparticle–gas plasma triple combination would be possible. Consequently, drug release speed could be more flexible by controlling the nanoparticle surface versus volume ratio [156]. Hence, nanoparticle–gas plasma combination could extend drug retention periods in vivo and enhance their intracellular concentrations [157]. Moreover, significant differences between various metal properties were found between Au and Ag nanoparticles combined with gas plasma [104,105]. For instance, gold nanoparticles are essentially non-toxic but notably enhance anticancer effects when combined with gas plasma exposure [97]. Similar findings were also made for nanoparticles coated with antibodies targeting FAK when combined with gas plasma treatment [99]. However, gold nanoparticles and gas plasma combination treatment also promoted the degradation of HER2/Neu and FAK1 [98], complicating potential therapeutic applications and mechanisms of action. At the same time, metals could be key to enhancing gas plasma-derived reactive species and anticancer toxicity via, e.g., the Fenton reaction [158]. For instance, iron-oxide magnetic nanoparticles were found to promote EGFR downregulation via suppressing pERK and pAKT in synergy with gas plasma exposure involving enhanced •OH generation catalyzed by Fe^2+^/Fe^3+^ [108]. As both pathways are essential for cancer survival and metastasis [159,160], targeted approaches combining nanoparticles and gas plasma exposure might be promising, depending on the tumor type and location. More in vivo studies are required to substantiate such concepts scientifically. Current nanoparticle combinations with gas plasma can be classified into two classes: metallic nanoparticles and nanoparticle-mediated drug delivery. It is also worthwhile to explore the synergy between non-metallic nanoparticles and gas plasma in future studies.

### 3.2. Local Treatment Modalities 

Several local treatment modalities, many of which are based on principles from physics, can be combined with gas plasma therapy [161]. A selection of such approaches is given in the following, with some showing promising results in vitro and in vivo (Table 2).

#### 3.2.1. Radiotherapy

Radiotherapy is an anticancer treatment that utilizes, e.g., X-rays, gamma rays, and neutrons. Radiotherapies are classified into external beam radiation therapy (EBRT) and internal radiation therapy. Radiotherapy directly induces DNA damage through ionizing radiation (IR) or indirectly promotes molecules adjacent to DNA to take up high-energy, thus generating reactive oxygen species, damaging DNA [162]. This involves, for instance, X-ray repair cross-complementing 1 (XRCC1) [163], p53 transcription factor, and genomic instabilities [164]. With the widespread application of radiotherapy, radio-resistance has emerged as a principal barrier [163]. It was proposed that radio-resistance is related to multiple factors, including altered DNA damage repair (DDR) regulatory molecules, cell cycle redistribution signalings, apoptosis escape, tumor microenvironment, cancer stem cells (CSCs), metabolic reprogramming, exosomes, and ferroptosis [165]. Additionally, normal tissue is also being influenced by high-dose irradiation rays, which leads to acute and late side effects [166], suggesting a need for reduced radiotherapy doses when the therapy is combined with other treatments. Recent research combined gas plasma with radiotherapy in murine melanoma cells, showing that the sequence of the treatments matters and that gas plasma exposure may sensitize tumor cells to radiotherapy and render them more immunogenic [116]. Enhanced toxicity was also found in breast cancer cell lines exposed to combined gas plasma and radiation therapy [117]. Interestingly, immortalized normal prostate cells would be less affected than prostate cancer cell lines in gas plasma-treated liquid and radiotherapy combination treatment [167]. This indicates that gas plasma could serve as one of the potential choices for radiotherapy amelioration in minimizing irradiation dosage and circumventing radio-resistance in the future, provided that ways are found for gas plasma to reach in-patient tumor sites. Few in vivo studies have reported on the relationship between combined gas plasma and radiation application. One was on melanoma, suggesting combined effects to be based on ROS generation, cell cycle arrest, and apoptosis induction involving radiation-derived DNA damage [6]. In general, gas plasma and radiation share common features, such as ROS production from intracellular and extracellular environments [168,169]. From a redox chemistry perspective, it has been suggested that gas plasma displays a similar reactive species chemistry pattern to X-rays [170]. Gas plasma could also be used not only to combine with radiotherapy for anticancer effects but also to contribute to tissue healing due to the radiotherapy-induced damage in non-tumor tissue [171]. In addition, radiotherapy was suggested to provide stimuli for enhanced efficacy of anticancer vaccines [172], which could also be achieved using gas plasma-derived reactive species production [173,174]. More in vivo studies and clinical data are awaited to verify or falsify the hypothesized promising nature of combined radio- and gas plasma therapy.

#### 3.2.2. Pulsed Electric Fields (PEFs) and Electrochemotherapy (ECT)

In 1991, electrochemotherapy (ECT) was first introduced into clinical use [175]. In ECT, pulsed electric fields (PEFs) are applied throughout the tumor tissue, thus enhancing membrane permeability to facilitate drug penetration into the cell [176]. Electrochemotherapy usually employs bleomycin and cisplatin [177] and is mainly implemented in treating superficial tumors. Mechanistically, ECT generally comprises toxicity in tumor cells and reflexive vasoconstriction triggered by high-voltage pulses [178]. However, restricted by PEFs, ECT is considered below temperatures for ablathermia to avoid heating injury. Chemotherapy concentrations required for anticancer effects can be reduced up to 50-fold when using ECT [179]. Recently, gas plasma was combined with electrochemotherapy against melanoma growth in vitro and in vivo, suggesting that combined applications are superior to single treatment [91]. Conjoined with ECT, gas plasma-treated PBS is also effective in LPB sarcoma cell-implanted mice [92]. The presumed mechanisms of action are numerous, including partially overlapping mechanisms of cell apoptosis, enhanced membrane permeability induced by lipid peroxidation, intracellular calcium increase by PEF, and intracellular ROS concentration elevation by gas plasma [180]. Similar to the microsecond PEF (μsPEF) in ECT, a more intensified nanosecond PEF (nsPEF) can stimulate extra- and intracellular ROS production [181,182] like gas plasma, which was found to act synergistically with gas plasma exposure in vitro [183]. Reports on the clinical combination of both medical technologies in cancer patients are yet to be awaited to drive this scientific concept further.

#### 3.2.3. Photodynamic Therapy

Photodynamic therapy (PDT) usually adopts photosensitizers to modify oxygen using external light and to release reactive species toward tissue, including tumors [184]. PDT can be sorted into acute and fractionated PDT based on treatment patterns. Its anticancer effect can be attributed to photodynamic reactions. Photodynamic reactions involve type 1-generating O_2_^•−^ and type 2-producing ^1^O_2,_ essential in anticancer effects [185]. These mechanisms include direct tumor cell destruction by inducing apoptosis and necrosis, tumor-supply vessel impairment from coagulation, and immune response activation [186]. Owing to photosensitizer dark toxicity [187] and dependency on the aerobic nature of the TME [188], some studies consider gas plasma to combine with PDT to avoid shortcomings, such as PDT resistance [189]. Gas plasma PDT integrating with polymeric nanoparticles showed enhanced anticancer effects in cervical cancer cells [7], and these improved results are also observed in lung cancer cells [114] and melanoma cells [112]. This synergy could be attributed to several factors. At first, gas plasma could be a light source to activate photosensitizers [112]. It was demonstrated that PDT could be stimulated by the overlap between gas plasma emission spectrum peaks and photosensitizer absorption spectrum peaks [190]. In this case, it is necessary to balance the duration of gas plasma irradiation, intensity, and wavelength range of various gases to comply with the photosensitizer type. Secondly, gas plasma and PDT would superimpose into a doubled-ROS-effect, and gas plasma-derived ROS/RNS could expand the toxicity onto tumor cells. Moreover, gas plasma could decrease PDT dependency on oxygen due to its exogenous property and tissue oxygenation improvement [191,192]. Lastly, PDT-mediated thermal effects could increase tumor cell membrane permeability, thus fostering more gas plasma-derived ROS to penetrate cancer cells [113]. In summary, current research lays the foundations for the joint usage of gas plasma and PDT, but solid in vivo trials are scarce to further substantiate the approach. Promising inventions like oxygen-independent PDT will provide more possibilities for clinical and research applications, also when combined with gas plasma exposure.

#### 3.2.4. Hyperthermia

Hyperthermia is one medical treatment to eradicate tumor tissue by adopting above-body temperature and classified into long-term low-temperature (~40–41 °C) and moderate-temperature (~42–45 °C) hyperthermia [193]. Hyperthermic intraperitoneal chemotherapy (HIPEC) is a commonly used anticancer treatment that lavages the abdominal cavity with heated chemotherapy drugs. When the temperature is above 37 °C, cell membrane fluidity increases, and its permeability and cytoskeleton arrangements are affected, hindering tumor cell movement, intracellular signal transduction, and tumor growth and metastasis [194]. Nevertheless, hyperthermia is rarely used alone and is usually combined with other therapies, and it was previously suggested that it could be combined with gas plasma-treated (oxidized) liquids [49]. Currently, gas plasma is regarded as an effective means to integrate with hyperthermia and display a profound synergistic effect. In U937 cells, gas plasma promoted apoptosis through cytoskeleton changes caused by hyperthermia [195]. A similar effect was also found in 3D bladder tumor spheroids [196]. Moreover, gas plasma-generated H_2_O_2_ was found to decrease TRPM2 membrane receptor activation thresholds by heat from hyperthermia to induce cell death [93]. These in vitro experiments indicate the joint usage possibility of gas plasma and hyperthermia, both on how to generate gas plasma and achieve hyperthermia, but in vivo studies are awaited to unravel the full potential of this combination.

#### 3.2.5. Hydrosurgery

**Figure 2:**
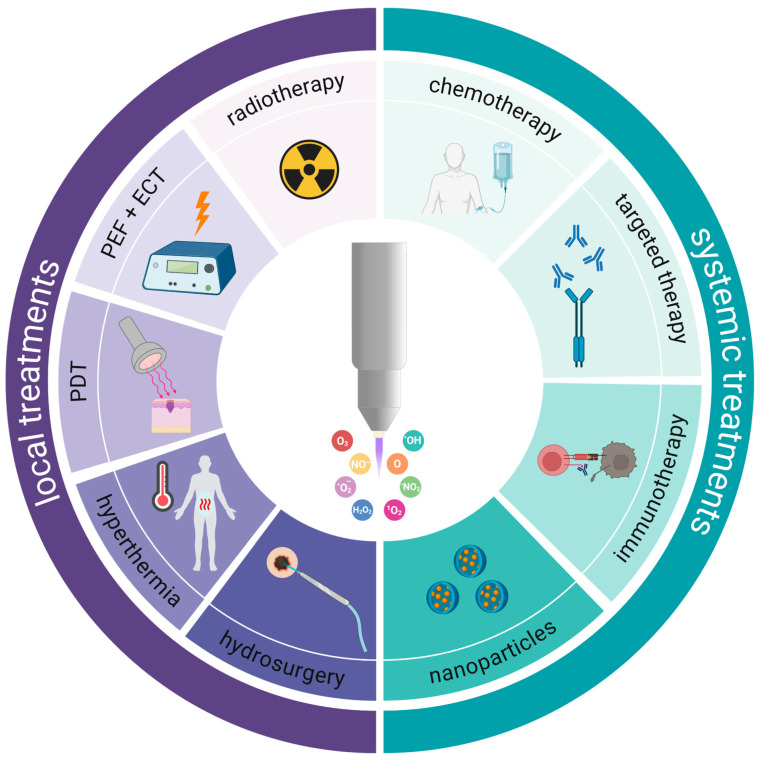
Potential combination treatments using medical gas plasma technology.

When conventional surgery is combined with gas plasma, such a combination is regarded as a sequential regimen. Therefore, the ideal surgery type that can serve as a combination therapy with gas plasma is hydrosurgery. Hydrosurgery uses high-pressure saline (0.9% sodium chloride) jets that cut tissue with less thermal stress compared to electrosurgical approaches and can be used to remove tumors via pure, pulsed, and abrasive modes [197]. Hydrosurgery has been shown to have tissue-selective separation effects for nerve and vessel protection due to their intrinsically high proportions of collagens and elastic fibers [198]. However, in some cases, hydrosurgical cutting was observed postoperatively with enhanced tissue adhesion. Here, gas plasma-treated cell culture medium (also sometimes referred to as plasma-activated medium, PAM) has been suggested to modify the adhesion properties of cells [199]. In addition, it was demonstrated that a plasma-treated cell culture medium could reduce the expression and secretion of pro-adhesive cytokines and extracellular matrix proteins in peritoneal fibroblasts [200]. Moreover, A431 cell adhesion properties were also inhibited by an oxidant-loaded cell culture medium, showing downregulation of CD44, hyaluronan synthase 2 (HAS2), HAS3, and hyaluronidases [201]. Generally, approaches using gas plasma-treated cell culture medium are experimental and not clinically relevant. However, this can be increased using liquids approved for humans, such as sodium chloride and Ringer’s lactate [49]. Along such lines, gas plasma-treated water (PTW) might alleviate postsurgical pain [202], promote excision healing, and avoid relapse of residual tumor tissue on margins [48]. There are two issues to be resolved: one is to find the most appropriate liquid and gas plasma source types, and the other is the flow speed effect in operation in hydrosurgery. Nevertheless, the combination of gas plasma with hydrosurgery holds great potential and may produce an underlying anticancer effect. All these mentioned combination treatments can be envisioned to be used together with medical gas plasma technology (Figure 2).

## 4. Bridging the Gap from Gas Plasma Medicine to Oncology in Patients

**Figure 3:**
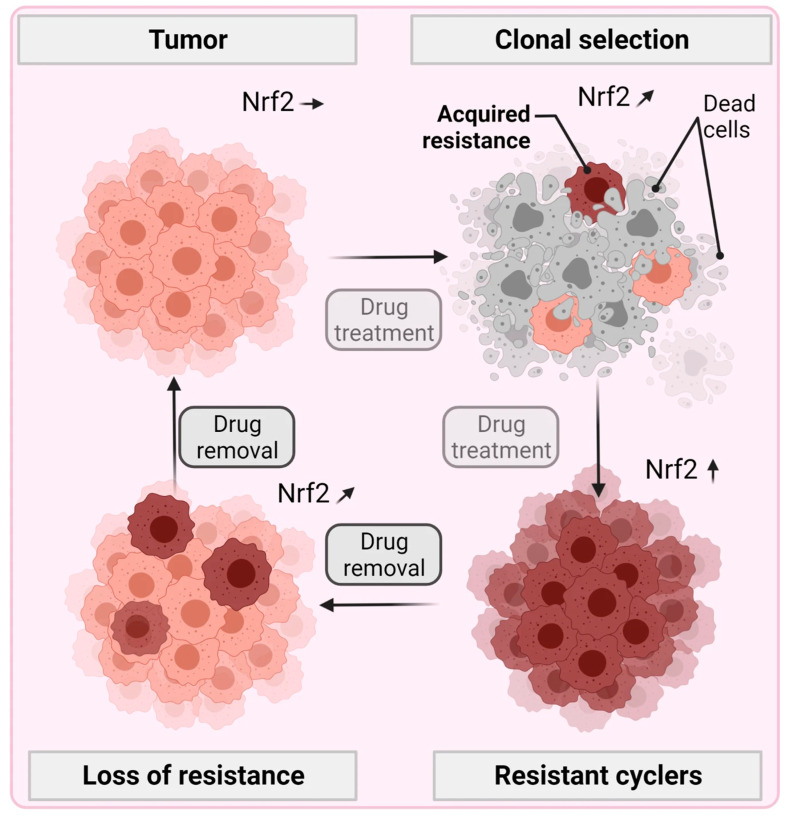
Potential mechanism of action on clonal selection through oxidative stress. Image is adapted from ref. [209].

**Figure 4:**
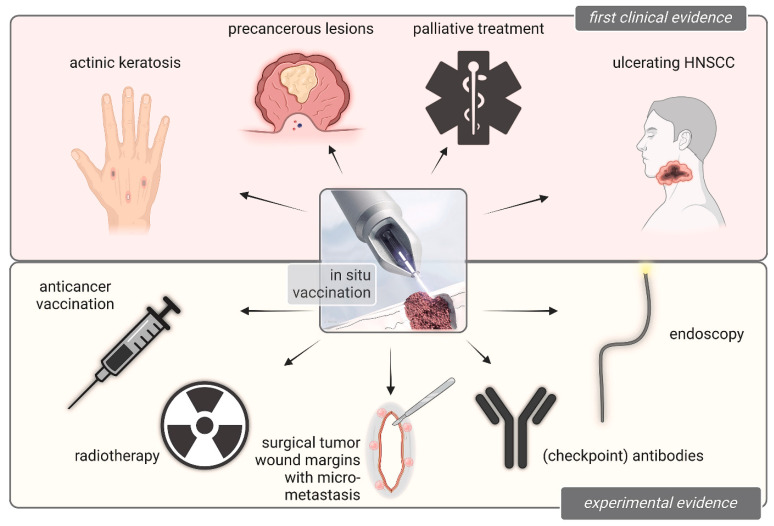
Current and future approaches to use and combine medical gas plasma technology in oncology. Image is adapted from ref. [5].

The number of experimental publications on gas plasma cancer treatment has increased recently, whereas clinical translational research has progressed only slowly [5]. This may have been due to gas plasma heterogeneity related to the application form (DBD [203], Jet [204]), working gas (helium, argon, air), air humidity [24], and many other factors [205]. In addition, there is the notion that gas plasma therapy may be most effective when combined with other local or systemic treatment modalities, such as immunotherapy [5]. Moreover, so far, medical experiences in gas plasma therapy against invasive tumors mainly pertain to the palliative setting [136]. Moreover, the treatment should be designed individually, depending on multiple factors, e.g., staging, tumor biology, mutational load, immune status, and the patient’s fitness. In addition, it is conceded that oxidative stress and related signaling, e.g., via Nrf2 (Figure 3), are prime hallmarks of gas plasma cancer treatment [5]. Despite some promising in vitro and in vivo results, the limited penetration depth [206] of gas plasma presumably requires repeated therapy of solid tumors [20,207], which would qualify skin cancer well for this therapy (Figure 4). Nevertheless, more than 100 known cancer entities exist. In the following, we will review potential oncology gas plasma applications to the deadliest cancers [208].

### 4.1. Melanoma

With increased depth, the skin layers comprise the epidermis, dermis, and subcutaneous tissue. In general, skin cancers originate from corresponding cellular sites like stratum basale-derived melanoma, basal cell cancer, and stratum spinosum-derived squamous cell cancer. This section will focus on melanoma; its conclusion could be a referential recommendation for other skin cancer types. At an early stage, surgical resection is considered the first-line treatment. As cancer progresses, it is hard to achieve complete resection (R0), limited by cosmetic or functional factors [210]. To reduce residual tumors, it is inevitable to introduce multiple therapies. At the same time, surgical removal of melanoma could be followed by intraoperative gas plasma treatment of tumor resection margins to reduce the risk of relapse. Molecularly, melanoma progression relates to PI3K/AKT and MAPK/ERK pathways [211]. Thus, downstream BRAF and MEK protein-blocking agents are recommended as adjuvant therapies with surgery to eradicate lesions. Even if chemotherapy, immunotherapy, and targeted therapy are applied as complementary means, drug resistance and side effects frequently occur in melanoma [212]. Gas plasma was previously suggested as an adjuvant experimental melanoma treatment. For instance, gas plasma was shown to induce G361 melanoma cell autophagy when combined with silymarin nanoemulsion. This stimulated PI3K/mTOR and EGFR pathways [109] and inhibited proliferation via HGF/c-MET regulation [110], which indicates that gas plasma shares a common signaling pathway with the above treatments. In addition, combined with doxorubicin, gas plasma augmented the anticancer effect synergistically [62]. Another set of studies indicated that the AhR pathway is activated under gas plasma exposure when combined with indirubin derivatives [132]. Moreover, we could previously show that, in B16-F10-bearing C57BL/6 mice, argon gas plasma exposure potently combined with imiquimod, a Toll-like receptor (TLR) 7 and 8 agonist [173]. Moreover, we also showed the combination treatment effects of gas plasma exposure against these melanoma cells with radiotherapy [116].

### 4.2. Lung Cancer and Bronchial Carcinoma

Small cell (SCLC) and non-small cell (NSCLC) are the primary lung and bronchial carcinoma categories. Owing to malignancy discrepancy, chemotherapy combination regimens are often considered in all stages of SCLC, usually based on platinum (carboplatin or cisplatin) or etoposide [213]. At the same time, NSCLC requires surgery, radiation, or chemotherapy as first-line therapy at early stages 1–3. Metastatic stage 4 recommendations relate to platinum-based chemotherapy or immunotherapy, such as gefitinib, used against cancers with activated EGFR mutations [214]. Lung cancer etiology generally involves multiple mechanisms, like ALK (anaplastic lymphoma kinase) gene rearrangement, and current treatments are based on such tumor-specific changes. Typically, little research is available on gas plasma treatment against lung cancer. In vitro, studies suggested that the anticancer effects of gas plasma may be associated with autophagy [86] and EGFR signaling [108], and only relatively few gas plasma studies focus on NSCLC in in vitro models yet. Moreover, combining gas plasma and recommended therapies in in vivo models lacks experimental proof. Song and colleagues [215] introduced plasma-treated liquid by oral gavage to tumors and suggested that drinking this liquid can reduce the lung cancer growth of tumors with partially extreme sizes. Oral administration routes are unusual for lung cancer treatment. Even though the author team reports on anticancer effects, the findings should be interpreted with care, as there is no logical explanation for how the gas plasma-derived reactive species can be retained if taken up into the highly antioxidative bloodstream from the colon without being degraded in the liver (first-pass effect) for subsequent delivery to the lung tissue. More feasible would be a gas plasma implementation via nebulized plasma-treated liquid or bronchoalveolar lavage (BAL) to target lung cancer.

### 4.3. Colorectal Cancer

Colorectal cancer can originate from the colon and rectum. In colon cancer, two major types of surgeries (colectomy and colostomy) are recommended, and adjuvant therapy includes chemotherapy or immune checkpoint inhibitor therapy as determined by biomarkers such as mismatch repair deficiency (dMMR). Likewise, surgery is preferable in rectal cancer, and chemotherapy with FOLFOX or CAPEOX is recommended as first-line treatment in the metastatic stage [216]. RAS, BRAF, HER2, and POLE/POLD-1 mutations could guide treatment choices in colorectal cancers that have not been resected. Several studies have suggested the role of gas plasma in colorectal cancer treatment. For instance, gas plasma elevated intracellular ROS to induce cell death, presumably via upregulation of Nox2 [217]. Moreover, gas plasma induced apoptosis via TRAIL and its death receptor 5 (DR5) in TRAIL-resistant colorectal cancer cells [218]. Combined with PDT [113,190], synergistic anticancer effects have been demonstrated in HT-29 cells. Single gas plasma exposure was suggested to involve mitochondrial pathways, which are key to its effects [219]. Nevertheless, no in vivo studies are available where colorectal cancers were treated directly at their appearance site (i.e., on the colon in an orthotopic model). Similarly, clinical experience of gas plasma exposure in colorectal cancer patients is not reported. As opposed to direct gas plasma exposure, it was indicated a few years ago that the peritoneal lavage of gas plasma-treated liquids [49] could effectively reduce metastatic colorectal cancer [43]. This was found for different, clinically employed liquids and is mechanistically mainly based on the presence of H_2_O_2_ in such liquids [220,221]. Another option would be employing gas plasma devices fitting into free endoscope channels, allowing gas plasma exposure inside the body. While technical concepts for such devices have been published [222], experimental in vivo anticancer effects in colorectal tumor models have not. Along similar lines, there are no reports on in vivo combination gas plasma treatment with pharmacological or immunotherapeutic intervention.

### 4.4. Pancreatic Cancer

Pancreatic cancer therapies list surgical resection as a preferred option [223]. First-line and second-line treatments are integrated into advanced or metastatic stage regimens based on body status rated by the Eastern Cooperative Oncology Group (ECOG) score system. For late-stage patients, FOLFIRINOX (5-fluorouracil, folinic acid, irinotecan, and oxaliplatin) and gemcitabine plus nab-paclitaxel are regarded as the mainstream choice [224] but are limited to the deadliest form of pancreatic cancer, PDAC (pancreatic ductal adenocarcinoma), having a poor prognosis [225]. Recently, oxidative stress [226] has been put forward as a new therapeutic target in PDAC, so gas plasma, a new and pleiotropic ROS source, has been tested in different pancreatic cell models. Hattori and colleagues [227] revealed that gas plasma-treated cell culture medium could activate caspases 3 and 7 and have a lethal effect on PDAC cell lines compared with normal pancreatic cells. Furthermore, gas plasma-treated PDAC cells were found to have altered inflammatory profiles and interfered with macrophage cluster formation in RAW 264.7 [228], related to the tumor-associated environment (TME) [229]. In addition to macrophages, tumor-supportive pancreatic stellate cells (PSCs) with immunosuppressive function may interfere with the toxicity and immunogenicity of gas plasma-treated PBS [230]. Gas plasma-treated cell culture medium was also found to reduce peritoneal carcinomatosis of PDAC effectively in vivo and supported the influx of immune cells into the tumor [231,232]. One of the few studies that combined gas plasma exposure (with treated Ringer’s lactate) with chemotherapy (cisplatin and gemcitabine) was tested in four pancreatic cancer cell lines in vitro and in ovo (a semi-in vivo model [233]), showing sound combined anticancer effects [75]. Notably, we thoroughly studied the metastasis risk effects of gas plasma-treated pancreatic cancer cells in different models and could not identify any increased risk due to the treatment [234].

### 4.5. Breast Cancer

Breast cancer treatments rely on multidisciplinary coordination, and corresponding therapies have evolved in diverse directions, including surgery, radiation, chemotherapy, immunotherapy, endocrine therapy (e.g., estrogen-receptor-directed tamoxifen) [235], HER2-directed trastuzumab [236], and biological therapy. Notwithstanding, the 5-year survivorship in metastatic type and triple-negative breast cancer (TNBC) is 26% [237] and 12% [238], respectively. In addition, drug-resistance issues emerge, rendering several therapies partially inefficient in the course of the treatment [239]. In such transition stages, metastasis, TNBC, and drug resistance are perceived as core issues in developing new therapies. For breast cancer, radiotherapy was previously suggested to induce abscopal effects [240]. Similarly, gas plasma exposure has been demonstrated to induce abscopal effects in mice that carried syngeneic 4T1 tumors on both flanks, which was accompanied by an enhanced influx of T-cells, such as T_H_17, suggesting elevated anticancer immunity via gas plasma exposure [9]. Besides direct gas plasma treatment of tumors, a previous study also found reduced postsurgical tumor recurrence if the resection margins of the primary tumors were exposed to gas plasma [241]. For TNBC, gas plasma was associated with a selective anticancer effect, presumably via hyperactivation of MAPK/JNK (mitogen-activated protein kinase/c-Jun NH 2-terminal kinase) and NF-κB (nuclear factor k-light-chain enhancer of activated B-cells) pathways [242]. Another study suggested gas plasma treatment in TNBC leads to downregulated BCL2A1 (Bcl-2-related protein A1) expression [243]. At the same time, one report speculated that gas plasma susceptibility relates to the expression of ER/PR (estrogen receptor/progesterone receptor) on HER2-positive breast cancer cells [244]. Notably, gas plasma was suggested to decrease breast cancer cell drug resistance [245]. Specifically, it was revealed that gas plasma recuperated paclitaxel-resistant MCF-7 cell sensitivity to chemotherapy via regulating drug resistance-associated genes. 4T1 breast cells also showed improved doxorubicin uptake levels when exposed to gas plasma [246]. In addition, gas plasma treatment was suggested to modify breast cancer stemness by regulating the AQP3/FOXO1 axis and inducing cell apoptosis through ERK inhibition and activation of p38-MAPK based on adulteration from HO/H_2_O_2_ [247]. Despite the above inspiring research, there is still very little known about effective in vivo combination treatments of anti-breast cancer drugs with gas plasma exposure. In contrast, studies on using gas plasma directly in breast cancer patients are absent. The only report concerning the latter pertains to a patient trial to prevent radiation-induced damage in the skin of breast cancer patients within an intrapatient-randomized, double-blinded, placebo-controlled trial to reduce radiation-mediated side effects rather than treating the cancer [171,248]. Of note, we were the first to recently test patient-derived breast cancer tissue exposed to gas plasma ex vivo, demonstrating increased cytotoxicity [249,250], which holds some promises for future research.

### 4.6. Prostate Cancer

Prostate cancer treatments are categorized into the risk group or staging system. The former adopts active surveillance as the first option [251]. The latter consists of localized and metastatic types, or early-stage and advanced-stage. Surgery and radiation therapy are preferred for early-stage prostate tumors, and hormone therapy combined with non-hormonal therapy is more favored for advanced stages. With the hormonal therapy resistance rate increasing along with side effects like postoperative urinary incontinence or erectile dysfunction [252] and androgen deprivation therapy (ADT)-associated osteoporosis [253], it is required to pursue alternative and low-invasive treatments for long-term survival of prostate cancer patients. A previous study suggested that DNA structure, cell viability, and colony-forming ability are debilitated by gas plasma, followed by necrotic death in BPH-1 and PC-3 cell lines, which primarily verifies the feasibility of gas plasma application on prostate cancer [254]. Subsequent parameter optimization achieved selective antitumor effects [255] associated with G0/G1 cell cycle arrest via activation of MAPK and NF-κB pathways [256]. However, no clinical efforts have been published on this topic so far. The crucial point is how gas plasma successfully targets the lesion and what dosage would be maximally effective at minimal invasiveness. At present, it is speculated that gas plasma could reach the prostate through a cryoprobe-like device based on transrectal ultrasound-guided percutaneous radical (TRUS) cryosurgical ablation [257]. Following this concept, more surgical approaches could serve as references to place gas plasmas inside the prostate and other cancer tissues, such as transurethral resection of the prostate (TURP) and high-intensity focused ultrasound (HIFU).

### 4.7. Urothelial (Bladder) Cancer

Bladder cancer is, with more than 550,000 new cases annually, among the second most frequent malignancies of the urogenital tract [258]. Sophisticated diagnosis and stage-adapted treatments are required to treat bladder cancer. Due to its high tumor mutational burden (TMB) (fourth highest average mutation rate among cancers), multimodal treatments are usually required, including surgery, (cisplatin) chemotherapy, and immune checkpoint inhibitors [259]. Regardless of the treatment, about one-third of the patients develop aggressive, muscle-invasive bladder cancer with a metastatic risk and mortality, leading to a five-year survival of less than 50% [260]. Almost all bladder cancers are urothelial carcinomas (also called transitional cell carcinoma), with the latter originating generally from the urethra, bladder, ureters, renal pelvis, and some other organs. Treatment side effects and poor prognosis required novel therapy approaches, and gas plasma exposure was tested in several studies as a potential new treatment modality. In vitro, gas plasma treatment was shown to induce urothelial cancer cell death, regardless of the plasma jet source operated by, e.g., argon [261], helium [77], or gas plasma-treated cell culture medium [262,263]. It has been noted that gas plasma exposure modifies the intracellular glutathione levels and mitochondrial function of bladder cancer cells [261,264]. It was also found that epithelial-to-mesenchymal transition fostered the sensitivity of bladder cancer cells to gas plasma exposure [265]. With regard to combination treatments, there has been a promising in vitro study combining hyperthermia with gas plasma-treated liquid to reduce the growth of 3D bladder cancer spheroids [196], while argon plasma jet exposure also reduced bladder cancer spheroid growth [261]. Another striking study combined gas plasma bladder treatment with four different antitumor drugs (cisplatin, methotrexate, adriamycin, and paclitaxel), identifying partially promising additive anticancer effects of the combination therapy [77]. In vivo, a decline in bladder cancer growth was observed when employing gas plasma-treated saline [266]. In vivo combination treatments with gas plasma exposure are not documented. Notably, we have recently investigated, for the first time, patient-derived urothelial cancer tissue exposed to a clinically approved (medical device) atmospheric pressure argon plasma jet ex vivo, finding more apoptosis and altered gene expression signatures from transcriptomic analysis [261]. Concerning the application of gas plasma, gas plasma-treated liquids that could be used to flush the urogenital tract could be a viable option, perhaps in combination with chemotherapy or targeted therapy. In addition, endoscopy is frequently used in urology and gynecology, and local bladder cancer tissue could be directly treated with endoscopic gas plasma devices, which have already been partly developed [267].

### 4.8. Liver Cancer and Malignancies of Intrahepatic Bile Duct

Hepatocellular carcinoma (HCC) and intrahepatic cholangiocarcinoma (ICC) are regarded as the common types of liver cancer. Early-stage tumors require surgery as the first-line treatment option, while systemic therapy is indicated as the recommended treatment with disease progression. Best supportive care, including palliative care, is the last resort for patients who are diagnosed with liver cancer in the advanced stage. Recently, liver cancer and cholangiocellular carcinoma have been increasingly studied in the context of plasma medicine. For instance, it has been shown in vitro that SK-HEP-1 tumor cells more easily detach following gas plasma treatment when compared to non-malignant cells [268] and that enhanced intracellular ROS were detected [269]. In addition, gas plasma-treated cell culture medium induced apoptosis in HepG2 to a higher extent than in a non-malignant cell line [270]. Similar findings were reported in other hepatic [269] and biliary cancer cells in vitro models [271]. Notably, gas plasma exposure was found to be toxic in both drug-sensitive and drug-resistant liver cancer cells [272] and gas plasma-treated medium potently combined with cisplatin in vitro [74]. In vivo anticancer effects of gas plasma exposure were recently shown in a cholangiocarcinoma mouse model, demonstrating reduced tumor size and growth [271]. Liver cancer still lacks in vivo gas plasma studies to support the general feasibility of the concept. The liver and gallbladder belong to intraperitoneal organs, and the bile duct opening is located at the ampulla of Vater, through which endoscopic gas plasma exposure would be feasible to reach, e.g., cholangiocarcinoma. This proof of concept was recently performed by adding a plasma catheter and successfully applying it to the porcine cholangiocarcinoma model [273]. Similarly, HCC gas plasma treatment improvement could be grounded on existing surgery techniques like radiofrequency ablation (RFA) [274]. In brief, gas plasma applications for hepatobiliary cancer are in their infancy. More preclinical experiments in combination with other treatments and the employment of sophisticated animal models are still needed, apart from the few in vitro studies.

### 4.9. Leukemia

Leukemia originates from blood or marrow and is classified into acute myeloid leukemia (AML), chronic myeloid leukemia (CML), acute lymphocytic leukemia (ALL), and chronic lymphocytic leukemia (CLL). Primarily, AML treatments are discussed, for they are the primary type in adults. Relevant treatments depend on the complex mutation category and consist of targeted therapy, chemotherapy, and corresponding side effects, like immune suppression accompanying them. In early studies, exposure time-related cytotoxic effects of different gas plasma devices were tested on leukemia cells, e.g., jets [275,276], DBD [277], and gliding arc discharge (GAD) [278]. Further research suggests that gas plasma can reduce leukemia cell viability in AML [279], induce cell apoptosis in CML [280] and ALL [281], and decrease metabolic activity in CLL [282], accompanied by changes in redox-related oxidative stress responses [283,284] as well as small RNA profiles [285]. Hence, there would be sufficient in vitro data in gas plasma experiments for future testing of this method using in vivo leukemia models. However, a significant hurdle is how gas plasma could target cells mainly located in a fluid (blood and bone marrow). As things stand, two strategies may be adopted for the clinical application of gas plasma against leukemia. The first is partially treating leukemia cells and re-injecting or infusing them into the blood circulation in the sense of a vaccine [174]. Yet, the TMB of leukemia is relatively low [286], and antigen accessibility of APCs and T-cells is not a significant obstacle in leukemia as a blood-based disease, indicating that this approach may not be too successful. The second scheme is to eliminate leukemia cells ex vivo. This idea has been around in the community for about ten years already, and, recently, the first study appeared to successfully gas plasma-treat primary leukemia cells from CLL patients’ blood [282]. The vision is to generate an extracorporeal circulation device equipped with gas plasma exposure to inactivate leukemia cells on the fly. Although, in principle, it is feasible, the central issue of this approach is twofold. First, we had previously investigated gas plasma-treated non-malignant (proband-derived) immune cells extensively. The main findings have been reviewed before [287] and can be summarized to (i) a very high susceptibility of all lymphocyte subpopulations (including T-cells, NK-cells, NKT-cells, and B-cells) to gas plasma exposure (although lymphocyte activation reduced toxic effects significantly) [288]; (ii) monocytes are generally more robust than lymphocytes, but both non-malignant lymphocytes and monocytes are significantly more sensitive to gas plasma-induced toxicity than their leukemia cell line counterparts [289]; (iii) myeloid and lymphoid leukemia cell lines are generally more sensitive to gas plasma-mediated toxicity compared to those of solid tumor origin, which exemplifies even more how sensitive primary immune cells are [290]; (iv) neutrophils generated extracellular traps (NETs) upon gas plasma treatment [291] that could pose a complication of gas plasma-treated leukemia-containing leukocyte apheresis products; (v) blood is a strong buffer for gas plasma-derived oxidants [292,293]; (vi) gas plasma exposure activates platelets, which could lead to thrombogenic reactions [294]; (vii) gas plasma potently leads to blood clotting (hemostasis) in murine [295] and human blood [296] independent of the gas plasma device used [297]; and (viii) no combination treatments of gas plasma with anti-leukemia drugs have been described so far that could further motivate any future clinical application. These potential adverse reactions including potential coagulation complications imply that much more research and gas plasma source fine tuning are required to facilitate gas plasma exposure against leukemia treatment.

### 4.10. Non-Hodgkin Lymphoma

Non-Hodgkin lymphoma (NHL) is one group of lymphocyte cancers originating from the lymph system (e.g., spleen). Given its heterogeneity, NHLs are classified into multiple subtypes requiring various treatment strategies. As the most common type, diffuse large B-cell lymphoma (DLBCL) therapies are mentioned and quoted as representatives to explain further the dilemma that NHL encounters. In DLBCL, various molecular differences and mutations determine corresponding subtypes. Therapeutic effects on 30–40% of DLBCL are still hindered [298], with hopes of relying on R-CHOP, which has been recommended as the first-line treatment. Other novel therapies are constantly designed on linker compound concepts like bispecific T-cell engagers (BiTEs) or antibody–drug conjugates (ADCs) for refractory types [299]. At present, no study explores the gas plasma anti-tumor effect on NHL. However, ROS have been studied in the field of NHL. Most studies reveal high ROS levels related to lymphoma pathogenesis and progression, including follicular lymphoma harboring higher peroxiredoxin levels associated with prolonged survival [300]. Mantle cell lymphomas show ROS exhaustion leading to apoptosis via interference with NADPH oxidase two expression [301]. In DLBCL, thioredoxin levels were found to be elevated, and its inhibition suppressed cell growth and modulated drug-resistant gene expression [302]. Moreover, GPX4 overexpression was shown to inhibit ROS-induced cell death and indicated poor prognosis in patients [303]. Given NHL subtype diversity and the absence of gas plasma-related studies, this cancer entity currently lacks promise through this experimental therapy modality.

### 4.11. High-Grade Glioma

Gliomas are a group of primary brain tumors that arise from neuroglial stem or progenitor cells and rank among the most common subtypes of this tumor entity [304]. Based on their considerable variation in the degree of malignancy, gliomas are categorized into low-grade (WHO grade 1–2) and high-grade (grade 3 and 4) tumors, with the latter showing higher recurrence rates and lower survival outcomes [305]. Although surgical resection is rarely curative as glioma cells extensively invade the surrounding brain parenchyma, it mitigates symptoms from tumor burden and enhances the efficacy of adjuvant therapies [306]. Since high-grade gliomas are highly heterogeneous in cytology and genetic signature, the clinical response of individual tumors may significantly differ, rendering treatment additionally challenging [307] and frequently causing therapy failures [308]. Therefore, strong research efforts are being made to define novel therapeutic avenues against GBM. Plasma medicine displays a bright prospect of innovative treatments in ongoing brain cancer research. First in vitro studies on using gas plasma technology were conducted more than a decade ago in T89G cells, showing jet plasma inhibitory effects on GBM growth and clonogenicity [309], which was suggested to be regulated via AKT1 and ERK1/2 signaling [310] in gas plasma-treated GBM cells. Gas plasma parameters can also be optimized towards GBM killing [311], which is effective against 3D GBM spheroids [312], and was indicated to reduce GBM migration and EMT [313]. Notably, several reports are available studying the combination treatment effects of gas plasma with anti-GBM drugs. For instance, TMZ-resistant glioma cells showed restored chemosensitivity following gas plasma exposure [71,314]. Gas plasma treatment also potently combined with TMZ cytotoxicity by inhibiting the GSH/GPX4 pathway [69]. Intriguingly, vitamin C showed a synergic anticancer effect with gas plasma-generated H_2_O_2_ against glioma [88]. Gas plasma was also suggested to increase the uptake of gold nanoparticles into GBM cells [106], providing synergistic toxicity [315]. Importantly, gas plasma-treated PBS and DBD treatment combined with auranofin induce ICD in GBM cells [87]. Thus, the gas plasma anticancer potential can be attributed to intrinsic cytotoxicity, drug sensitivity restoration, and synergy with guideline therapies. A recent study also suggested that electromagnetic fields produced by gas plasma devices contribute to anti-GBM effects [316]. Strikingly, we recently were the first to show in patient-derived glioblastoma tissue that apoptosis is elicited following gas plasma exposure [317]. There are several potential treatment modalities for employing gas plasma to treat brain tumors. First, gas plasma could treat the resection margins of surgically reduced tumors, which inactivates cancer cells and potentially induces ICD to promote anticancer immunity [318]. Second, gas plasma-treated liquids or hydrogels could be used to lavage tumor sites or section margins or be injected into the tissue. For the latter, intranasal delivery [319] with a gas plasma-treated pharmaceutical formulation could be an option. Potentially, intraventricular catheter systems, as Ommaya reservoirs, which are sometimes used for the administration of chemotherapeutics, could be used as a delivery route for gas plasma access. In general, while ample in vitro data is available [61,68,89,310,320], there is no convincing (orthotopic) in vivo evidence to support gas plasma-assisted brain tumor treatment. Nevertheless, given the high mortality of GBM and early data with patient-derived tumors, gas plasma remains a promising therapy modality.

### 4.12. Esophagus Cancer

Esophageal cancer is categorized into esophageal and esophagogastric cancers. Tumor treatment discussion focuses on the typical type of squamous cell cancer. Due to cancer’s proximity to cardiopulmonary structures, surgery will inevitably face more risk, and postoperative complications like dysphagia may occur. Once its progression proceeds into the metastatic stage, systemic therapies are the preferred option. A typical gas plasma application in esophageal cancer is argon plasma coagulation (APC, a hotter tissue-necrotic plasma)-assisted surgical removal [321]. However, only a few studies explored the efficacy and mechanisms of action of gas plasma exposure on esophageal cancer. Cold direct gas plasma treatment showed cytotoxic effects in KYSE-30 in vitro [322]. It was proposed [323] that enhanced ROS could inhibit cancer stem cells despite the cancer progression association with ROS. Other experiments also demonstrated the anticancer role of ROS on esophageal cells [324]. We recently provided the first evidence of ex vivo gas plasma exposure toxicity in patient-derived esophageal cancer [250]. Gas plasma application in esophageal cancer has several directions. Firstly, it could adopt the argon plasma coagulation (APC) device model and integrate itself into an entity by adjusting parameters. Secondly, plasma-treated liquid would be a relatively feasible means to contact cancer lesions orally, but more data are still needed to support this concept. Overall, esophageal cancer research on gas plasma should be combined with existing technical advantages.

### 4.13. Other Cancers

While this review has focused primarily on the deadliest cancers that have been thoroughly investigated regarding gas plasma combination therapies, it is essential to acknowledge that many other cancer entities—such as endometrial cancer, gastric cancer, hepatoblastoma, myeloma, ovarian cancer, sarcomas, and squamous cell carcinomas (SCC) (Table 2)—have been less frequently studied. Except for SCC, little preliminary research exists for these tumor types that examined the application of cold physical plasma in combination with a second treatment method. Especially rare sarcomas and pediatric tumors remain largely unexplored and lack research in the context of gas plasma and its implementation in multimodal treatment strategies. This gap highlights an essential field for future investigations, as expanding gas plasma applications across a broader range of malignancies could potentially improve therapeutic options. We therefore encourage further research efforts to explore the efficacy and mechanisms of gas plasma treatment in these underrepresented cancer entities.

## 5. Challenges in Gas Plasma Combination Therapy

Applied as monotherapy, gas plasma is considered a minimally invasive and well-tolerated treatment technique without severe side effects due to high tumor selectivity [18]. Accredited medical devices are routinely used in clinical dermatology [310]. However, safety assessments regarding its promising combinatory potential with other therapy options are entirely lacking, and in addition, several other challenges need to be addressed prior to clinical translation. First, it is necessary to define the most effective treatment regime, e.g., plasma application time point and duration, that exploits the optimal synergistic window for the simultaneous or sequential administration of additional therapies. For example, to harness the full potential of plasma-generated transient oxidation by the temporary burst of ROS/RNS, it is critical to exactly coordinate the treatment sequence and precisely tune treatment parameters. Thus, plasma treatment prior to radiation therapy exerted stronger antitumoral efficacy compared to the opposite sequence [116]. In a comparable context, plasma can mediate drug inactivation via oxidative modification [134]. Hence, the full spectrum of redox interactions between plasma-induced ROS/RNS and administered therapeutic agents or other therapy forms should be unraveled before clinical usage to avoid antagonism or efficacy loss. A third major obstacle is to elucidate which combination approach is rational for what kind of malignancy, as tumor entities demonstrated varying gas plasma sensitivities in previous studies [290]. For instance, combining gas plasma with a specific treatment modality may only be feasible in a few cancer types, as its combination with bleomycin evidently showed enhanced survival reduction in melanoma cells but no significant impact in glioblastoma cell lines compared with the drug monotherapy [61]. Lastly, these tumor insensitivities, either intrinsic or acquired, need to be extensively investigated to identify underlying mechanisms and potential ways to prevent or overcome these for an optimized treatment outcome. In this light, a previous study reported on the correlation between metabolic turnover rate and absolute expression levels of aquaporins and NOX members with gas plasma resistance [290]. Furthermore, molecular biological comparison of wild-type and plasma-resistant skin cancer cells revealed not only an adapted antioxidative capacity, increased G2-phase cycle arrest, and diminished pro-inflammatory profile but also diminished immunogenicity of plasma-resistant cancer cells. Besides markedly upregulation of immunosuppressive markers in vivo, transcriptome analysis showed strong and significant correlation of IL1R2 with the gas plasma resistance [325]. These defense mechanisms could also promote insusceptibility towards other tumor toxic treatments, emphasizing the urgent necessity for their imperative discovery.

## 6. Conclusions and Outlook

Only five completed studies noted in ClinicalTrials.gov explored gas plasma application on cancer or precancerous lesions (Table 1). Before proceeding with more clinical studies, for many tumors, it remains to be explored what combination of treatment modalities and gas plasma source types and parameters would be optimal to provide convincing effects in patients. A notion favoring the further investigation of medical gas plasma technology in oncology is that several existing therapeutic modalities involve ROS elevation, e.g., radiodynamic treatment (RDT) [326], sonodynamic therapy (SDT) [327], electrodynamic therapy (EDT) [328], and chemodynamic therapy (CDT) [329]. The anticancer effect might be intensified when gas plasma integrates the above treatments. A second aspect is integrating medical techniques with gas plasma, thus making gas plasma more accessible to cancer, which involves new administration methods such as endoscopy and gas plasma device reengineering. In general, it seems preferable to administer gas plasma along natural anatomical orifices unless there is an artificial opening like a stoma during colostomy. A third aspect is how gas plasma reaches the target: penetration depth, membranous entrance, and ROS half-life. It has been proposed that more than 80% RNS and less than 5% ROS can penetrate 500 µm tissue [330], and the penetration rate is less than 1% in 1 mm of tissue [331]. As another mechanism of action, gas plasma-derived reactive species may induce lipid peroxidation to enhance the entry of further reactive species [332,333]. It is also one of the main questions whether short-lived species produced by direct gas plasma treatment are required for tissue anticancer effects, or if long-lived species suffice [5,334]. Substantial research has successfully elaborated synergies of gas plasma with other therapies, and multiple mechanisms regulate such synergies. However, insufficient preclinical proof often bottlenecks synergistic gas plasma clinical translation in oncology, but the promising effects of gas plasma monotherapies in dozens of in vivo trials and a few clinical investigations generate hope for convincing future concepts and applications in gas plasma oncology.

## Figures and Tables

**Table 1:** Clinical trials registered on ClinicalTrials.gov that mention the utilization of medical gas plasma technology to treat cancer.

Type of Cancer or Precancerous Lesion	Status	Main Outcome Indicators	Devices	Trials No.	Search Term	Outcome
Cervical Intraepithelial Neoplasia (CIN) Grade III	Completed	Rate of histological complete remission	Non-invasive physical plasma device	NCT04753073	Cancer + Physical Plasma	-
Cervical Intraepithelial Neoplasia	Completed	Pathological remission of cervical intraepithelial neoplasia	Non-invasive physical plasma device	NCT03218436	Cancer + Physical Plasma	86.2% rate of full remission of CIN1/2 lesions [45]
* Surgical Margin and Macroscopic Tumor Sites	Completed	Number of participants with complications due to cold plasma application	Canady Helios cold plasma scalpel (CHCPS)	NCT04267575	Cancer + Cold Plasma	CHCP combined with surgery significantly, is safe and reduces local recurrence after Stage IV tumor removal [48]
Familial Adenomatous Polyposis (FAP)	Recruiting	Polyp number and size	Argon plasma coagulation (APC)	NCT06435533	Cancer + Cold Plasma	-
Skin Disorders (including Actinic Keratosis)	Completed	Clinical improvement upon reviewing photo-documentation	Custom-made nonthermal atmospheric plasma device	NCT02759900	Cancer + Cold Plasma	Out of 17 treated lesions, 9 were removed and 3 significantly improved [47]
* Peritoneal Tumor Tissue	Recruiting	Histology of resected tumor nodules	J-plasma	NCT06796634	Cancer + Cold Atmospheric Plasma (CAP)	-
Gastric Low-Grade Intramucosal Neoplasia	Recruiting	Complete ablation of gastric low-grade intraepithelial neoplasia	Hybrid argon plasma coagulation (APC)	NCT04197180	Cancer + Gas Plasma	-
Non-small Lung Cancer with Endobronchial Obstruction	Terminated	Treatment time until failure	Argon plasma coagulation (APC)	NCT03564054	Cancer + Gas Plasma	-
Anal Intraepithelial Neoplasia (AIN)	Completed	Absence of AIN at 24-month follow-up	Argon plasma coagulation (APC)	NCT00428285	Cancer + Gas Plasma	65% patients were clear of AINs; repeated APC treatment was needed; no serious adverse events [46]

* Unspecified cancer, - results not published at date of publication.
